# Synthesis and Cytotoxic Activity of Novel Indole Derivatives and Their *in silico* Screening on Spike Glycoprotein of SARS-CoV-2

**DOI:** 10.3389/fmolb.2021.637989

**Published:** 2021-05-11

**Authors:** Perumal Gobinath, Ponnusamy Packialakshmi, Kaliappillai Vijayakumar, Magda H. Abdellattif, Mohd Shahbaaz, Akbar Idhayadhulla, Radhakrishnan Surendrakumar

**Affiliations:** ^1^PG & Research, Department of Chemistry, Nehru Memorial College (Affiliated Bharathidasan University), Puthanampatti, India; ^2^Department of Chemistry, M. Kumarasamy College of Engineering, Karur, India; ^3^Department of Chemistry, College of Science, Deanship of Scientific Research, Taif University, Taif, Saudi Arabia; ^4^South African Medical Research Council Bioinformatics Unit, South African National Bioinformatics Institute, University of the Western Cape, Cape Town, South Africa; ^5^Laboratory of Computational Modeling of Drugs, South Ural State University, Chelyabinsk, Russia

**Keywords:** indole, Mannich base, cytotoxic activity, COVID-19, spike protein

## Abstract

This work investigated the interaction of indole with SARS-CoV-2. Indole is widely used as a medical material owing to its astounding biological activities. Indole and its derivatives belong to a significant category of heterocyclic compounds that have been used as a crucial component for several syntheses of medicine. A straightforward one-pot three-component synthesis of indole, coupled with Mannich base derivatives 1a–1j, was synthesized without a catalyst. The products were confirmed by IR, ^1^H-NMR, ^13^C-NMR, mass spectra, and elemental analysis. The indole derivatives were tested for cytotoxic activity, using three cancer cell lines and normal cell lines of Human embryonic kidney cell (HEK293), liver cell (LO2), and lung cell (MRC5) by MTT assay using doxorubicin as the standard drug. The result of cytotoxicity indole compound 1c (HepG2, LC_50_−0.9 μm, MCF−7, LC_50_−0.55 μm, HeLa, LC_50_−0.50 μm) was found to have high activity compared with other compounds used for the same purpose. The synthesized derivatives have revealed their safety by exhibiting significantly less cytotoxicity against the normal cell line (HEK-293), (LO2), and (MRC5) with IC_50_ > 100 μg/ml. Besides, we report an *in silico* study with spike glycoprotein (SARS-CoV-2-S). The selective molecules of compound 1c exhibited the highest docking score −2.808 (kcal/mol) compared to other compounds. This research work was successful in synthesizing a few compounds with potential as anticancer agents. Furthermore, we have tried to emphasize the anticipated role of indole scaffolds in designing and discovering the much-awaited anti-SARS CoV-2 therapy by exploring the research articles depicting indole moieties as targeting SARS CoV-2 coronavirus.

## Introduction

Coronavirus has proved to be the most deadly of the 21st-century epidemics by being responsible for emergent communicable disorders. It first manifested its presence through the onset of dangerous pneumonia, started by the (SARS-CoV) infestation in 2003 (Rahman et al., 2020). In December 2019, many lung fever patients infected by a novel coronavirus were announced in Wuhan, China (Chan et al., 2020; Li et al., 2020; Zhu et al., 2020). The SARS-CoV-2 has been the cause of greater than 1.27 million deaths as of November 11, 2020 (Lu et al., 2020; Wu et al., 2020; Zhou et al., 2020). The acronym for coronavirus, namely, SARS-CoV-2, was assigned by the World Health Organization (WHO) on February 11, 2020 (Gorbalenya et al., 2020). SARS-CoV-2 has become a global health crisis involving around 212 countries (World Health Organization, 2020). Several drug mixtures are still being used.

However, the remedial outcome has been meager with secondary response (Cao et al., 2020). Adenosine triphosphate (ATP) analog was used as an antiviral drug to counter the effects of COVID-19, but more statistics are required to demonstrate its efficiency (Cohen, 2020; Holshue et al., 2020; Wang et al., 2020). On August 11, 2020, Russia became the first nation to approve a vaccine (sputnik V) to protect against infection by COVID-19 (Talha, 2020). The inherent RNA of coronaviruses and its structure information is described and discussed by other researchers (Hussain et al., 2005; Chen et al., 2020). In the biorhythms of coronaviruses, some functional and non-functional proteins are involved (Ramajayam et al., 2011; Ren et al., 2013). The emergence of drug-resistance for antiviral activity and defective antiviral drugs stimulates a great demand to develop a less toxic and more potent antiviral agent. In this regard, researchers have recently focused on naturally available indoles and their derivatives.

**FIGURE 1:**
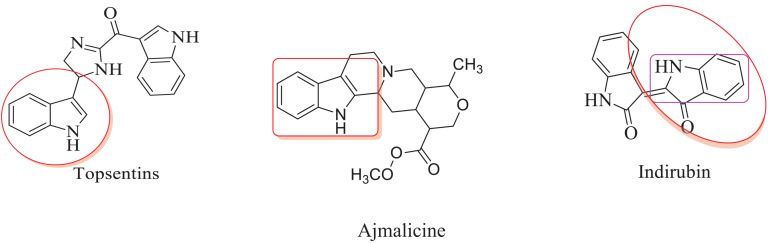
Natural products of indole derivatives.

**FIGURE 2:**
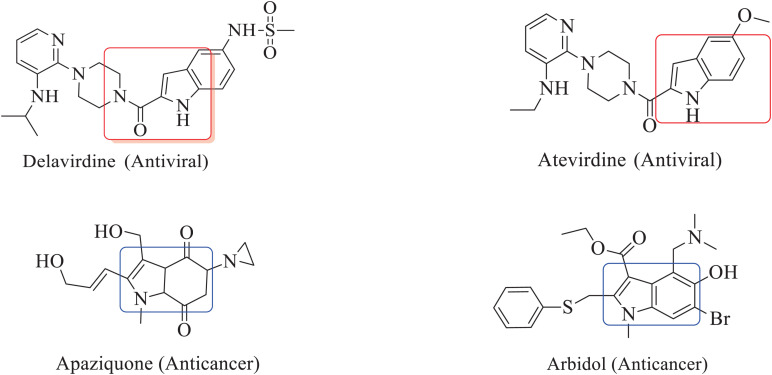
Biological activities of indole derivatives.

The inclusion of indole is the most significant structural modification in drug development, and it is labeled as one of the “privileged scaffolds” (Evans et al., 1988; deSa Alves et al., 2009; Welsch et al., 2010). The enlargement of a new technique for the pattern of C-N and C-C bonds that evade the pore functional group is tremendously significant in current organic chemistry (Ricci, 2008). Amino methylation is a crucial method for direct carbon–carbon and carbon-nitrogen bond-forming reactions (Hwang and Uang, 2002). Usually, amino methylation is done by the Mannich reaction using aldehyde as a methylene group source (Mannich and Krosche, 1912). Indole is perhaps the most ubiquitous motif in nature (Humphrey and Kuethe, 2006). Many natural and synthetic indole derivatives have been in great demand in medical and pharmaceutical applications since they can bind with high affinity to many receptors (Sundberg, 1970, 1996; Lounasmaa and Tolvanen, 2000; Horton et al., 2003; Gu and Hamann, 2005; Somei and Yamada, 2005; Shiri, 2012). Previously reported natural products of indole derivatives are shown in Figure 1 (Chen et al., 2019), and the biological activities of indole derivatives are offered in Figure 2 (Kumari and Singh, 2019). Indole regulates numerous aspects of microorganism physiology, including reproductive structure formation, body stability, resistance to medication, biofilm formation, and virulence (Chadha and Silakari, 2017). Based on the above properties, we prepared new indole derivatives 1a–1j via the Mannich reaction. As the indole compounds have been rigorously involved in ailments including viral infections and cancer, there exists a profound scope of exploring these multiple nuclei to curb coronaviruses (Zhang et al., 2015). Here we demonstrated that the indole moiety potently blocked the infectivity of SARS CoV-2 by targeting glycoproteins. They also potently block the enzymatic activity of SARS CoV-2 and replication of coronavirus (Hattori et al., 2021). Therefore, through this, indole derivatives developed against SARS-CoV-2 epidemics using *in vitro* and *in silico* approaches may be of immense value at this hour of global emergency and in the future.

## Experimental

### General

All the chemicals were purchased from Merck. The melting point was determined using an open capillary tube, and it is uncorrected. The IR spectra were recorded in KBr on a Shimadzu 8201pc (4000–400 cm^–1^). ^1^H and ^13^C-NMR spectra were recorded on Bruker Avance II NMR spectrometer 300 MHz with DMSO-d_6_ as solvent using tetramethylsilane (TMS) as an internal standard. Mass spectra were recorded using Clarus SQ8 (Perkin Elmer), and the elemental analysis (C, H, and N) was performed on a Varian EL III instrument.

#### General Procedure for the Synthesis of Compounds 1a–1j

We compounded Furan-2-ylmethylenehydrzine (0.01 mol), indole (0.01 mol), and substituted aldehydes (0.01 mol) in ethanol solution to give a yellow solution with light brown color precipitate. The residue was recrystallized with ethanol. The obtained compound was purified by thin-layer chromatography (TLC). Hexane was used as eluting and solvent in TLC. All the synthesized compounds were separated by column chromatography.

#### (*E*)-1-((2furan-2ylmethylene)hydrazinyl)phenyl)methyl) 1H-indole (1a)

Light yellowish brown solid: mp 250°C; IR (KBr) (cm^–1^) 3440 (NH str), 3080 (CH-str Ar-ring), 1623 (C = N), 1092 (N-CH-N). ^1^H NMR (DMSO-d_6_), δ (ppm) J (Hz): 8.41 (s, 1H, CH = N), 7.99–7.79 (d, 2H, indole), 7.82–6.70 (m, 3H, furan), 7.40–6.95 (m, 9H, Ar), 7.08 (s, 1H, NH), 7.08 (s, 1H, CH); ^13^C NMR (DMSO-d_6_) δ(ppm): 150.46, 145.73, 118.96, 113.43 (4C, Furyl ring), 143.52, 128.57, 127.78, 126.95, 126.90, 125.25 (6C, Ph ring), 136.25, 126.62, 125.23, 121.69, 120.09, 118.23, 111.57, 110.76 (8C, indole ring), 135.23 (1C, C = N), 40.59 (1C, N-CH-N); EI-MS (Relative intensity %): m/z 315.14 (M^++^, 20); Elemental analysis: *Anal*.C_20_H_17_N_3_O: C, 76.20; H, 5.45; N, 13.35; Found C, 76.25; H, 5.55; N, 13.28.

#### 1-((2furan-2ylmethylene)hydrazinyl)3-nitrophenyl) methyl)1H-indole (1b)

Light brown solid: mp 258°C; IR (KBr) (cm^–1^) 3435 (NH str), 3058 (CH-str Ar-ring), 1590 (NO_2_^–^), 1623 (C = N), 1094 (N-CH-N). ^1^H NMR (DMSO-d_6_), δ (ppm) J (Hz): 8.33–6.75 (m, 9H, Ar), 8.10 (s, 1H, CH = N), 7.95–7.82 (d, 2H, indole), 7.69–6.74 (m, 3H, furan), 7.09 (s, 1H, NH), 6.73 (s, 1H, CH); ^13^C NMR (DMSO-d_6_) δ (ppm): 150.62, 145.64, 118.96, 113.50 (4C, Furyl ring), 147.75, 135.85, 127.84, 126.69, 114.20, 114.12 (6C, Ph ring), 136.53, 126.62, 125.25, 121.62, 120.16, 118.25, 111.48, 110.70 (8C, indole ring), 135.27 (1C, C = N), 40.54 (1C, N-CH-N); EI-MS (Relative intensity %): m/z 360.12 (M^+^, 20); Elemental analysis: *Anal*.C_20_H_16_N_4_O_3_: C, 66.67; H, 4.49; N, 15.56; Found C, 66.62; H, 4.55; N, 15.48.

#### 1-((2furan-2ylmethylene)hydrazinyl)1H-indole-1-yl)methyl)phenol (1c)

Brown solid: mp 272°C; IR (KBr) (cm^–1^) 3585 (OH), 3449 (NH str), 3086 (CH-str Ar-ring), 1629 (C = N), 1091 (N-CH-N). ^1^H NMR (DMSO-d_6_), δ (ppm) J (Hz): 10.24 (s, 1H, OH), 8.52 (s, 1H, CH = N), 7.95–7.52 (d, 2H, indole), 7.68–6.97 (m, 3H, furan), 7.42–6.72 (m, 9H, Ar), 7.06 (s, 1H, NH), 6.74 (s, 1H, CH); ^13^C NMR (DMSOd_6_) δ (ppm): 176.36 (1C, Ph-OH), 150.25, 145.24, 118.42, 113.47 (4C, Furyl ring), 142.18, 129.48, 129.50, 114.25, 114.22 (5C, Ph ring), 136.04, 127.89, 125.24, 121.67, 120.15, 118.29, 111.52, 110.76 (8C, indole ring), 135.29 (1C, C = N), 40.51 (1C, N-CH-N); EI-MS (Relative intensity %): m/z 331.13 (M^+^, 20); Elemental analysis: *Anal*.C_20_H_17_N_3_O_2_: C, 72.50; H, 5.18; N, 12.69; Found C, 72.45; H, 5.24; N, 12.65.

#### 1-((4-chlorophenyl)2-furan-2ylmethylene)hydrazinyl) methyl)-1H-indole (1d)

Light brown solid: mp 260°C; IR (KBr) (cm^–1^) 3442 (NH str), 3082 (CH-str Ar-ring), 1626 (C = N), 1094 (N-CH-N), 818 (C-Cl). ^1^H NMR (DMSO-d_6_), δ (ppm) J (Hz): 8.11 (s, 1H, CH = N), 7.98–7.81 (d, 2H, indole), 7.84–6.74 (m, 3H, furan), 7.32–6.70 (m, 9H, Ar), 7.06 (s, 1H, NH), 6.74 (s, 1H, CH); ^13^C NMR (DMSO-d_6_) δ (ppm): 150.31, 145.35, 118.85, 113.40 (4C, Furyl ring), 136.10, 131.67, 130.50, 129.8, 129.59, 129.57 (6C, Ph ring), 136.25, 127.78, 125.26, 121.59, 120.10, 118.20, 111.59, 110.73 (8C, indole ring), 135.21 (1C, C = N), 40.50 (1C, N-CH-N); EI-MS (Relative intensity %): m/z 349.10 (M^+^, 20); Elemental analysis: *Anal*.C_20_H_16_ClN_3_O: C, 68.68; H, 4.62; N, 12.03; Found C, 68.65; H, 4.63; N, 12.05.

#### 1-((2furan-2ylmethylene)hydrazinyl)4-methoxyphenyl) methyl)1H-indole (1e)

Light yellowish brown solid: mp 275°C; IR (KBr) (cm^–1^) 3444 (NH str), 3056 (CH-str Ar-ring), 2854 (OCH_3_), 1618 (C = N), 1089 (N-CH-N). ^1^H NMR (DMSO-d_6_), δ (ppm) J (Hz): 8.22 (s, 1H, CH = N), 7.96–7.86 (d, 2H, indole), 7.62–6.80 (m, 3H, furan), 7.47–6.69 (m, 9H, Ar), 7.09 (s, 1H, NH), 6.75 (s, 1H, CH). 3.86 (s, 3H, OCH_3_); ^13^C NMR (DMSO-d_6_) δ (ppm): 158.62 (1C, Ph, OCH_3_), 150.24, 145.62, 118.94, 113.48 (4C, Furyl ring), 136.51, 126.61, 125.27, 121.60, 120.18, 118.28, 111.50, 110.72 (8C, indole ring), 135.83, 127.82, 126.67, 114.18, 114.09 (5C, Ph ring), 135.29 (1C, C = N), 55.74 (1C, OCH_3_), 40.54 (1C, N-CH-N); EI-MS (Relative intensity %): m/z 345.15 (M^+^, 20); Elemental analysis: *Anal*.C_21_H_19_N_3_O_2_: C, 73.04; H, 5.55; N, 12.16; Found C, 70.59; H, 5.60; N, 12.30.

#### 2-((2-furan-2-ylmethylene)hydrazinyl)(1H-indol-1-yl)methyl)phenol (1f)

Light brown solid; mp 265°C; IR (KBr) (cm^–1^) 3447 (NH str), 3089 (CH-str Ar-ring), 2915 (C-OCH3), 1628 (C = N), 1090 (N-CH-N). ^1^H NMR (DMSO-d_6_), δ (ppm) J (Hz): 9.82 (s, 1H, OH), 8.25 (s, 1H, CH = N), 8.22–6.83 (m, 9H, Ar), 7.97–7.81 (d, 2H, indole), 7.71–6.49 (m, 3H, furan), 7.19 (s, 1H, NH), 6.71 (s, 1H, CH); ^13^C NMR (DMSO-d_6_) δ (ppm): 148.90, 144.45, 119.08, 113.06 (4C, Furyl ring), 139.89 (1C, Ph, OH), 136.56, 126.79, 128.41, 121.07, 120.79, 119.82, 109.76, 100.24 (8C, indole ring), 131.01, 126.02, 120.42, 118.92, 116.05 (5C, Ph) 134.19 (1C, C = N), 40.49 (1C, N-CH-N); EI-MS (Relative intensity %): m/z 331.37 (M^+^, 20); Elemental analysis: *Anal*.C_20_H_17_N_3_O_2_: C, 72.50; H, 5.72; N, 12.69; Found C, 72.40; H, 5.51; N, 12.48.

#### 4-((2-furan-2-ylmethylene)hydrazinyl)1H-indol-1-yl)methyl)-2-Methoxyphenol (1g)

Brown solid: mp 289°C; IR (KBr) (cm^–1^) 3442 (NH str), 3082 (CH-str Ar-ring), 2910 (C-OCH3), 1626 (C = N), 1094 (N-CH-N). ^1^H NMR (DMSO-d_6_), δ (ppm) J (Hz): 10.48 (s, 1H, OH), 8.93 (d, 1H, J = 6.1 Hz, Furyl ring), 8.16 (s, 1H, methylene), 7.92–7.80 (d, 2H, *J* = 7.6 Hz, indole), 7.87 (d, 1H, *J* = 6.7 Hz, Furyl ring), 7.20, 7.18, 6.84, 6.75 (m, 4H, indole ring), 7.09 (s, 1H, NH), 6.64 (t, 1H, Furyl ring), 6.65–6.74 (m, 2H, Ar-CH), 6.71 (s, 1H, CH), 3.85 (s, 3H, OCH_3_) 3.50 (s, 1H, CH); ^13^C NMR (DMSO-d_6_) δ (ppm): 150.21, 145.38, 118.82, 113.41 (4C, Furyl ring), 147.52, 146.82, 142.30, 126.37, 120.18, 115.39, 56.09 (7C, Ph ring), 136.15, 127.79, 125.23, 121.49, 120.00, 118.28, 111.52, 110.71 (8C, indole ring), 135.22 (1C, C = N), 40.51 (1C, N-CH-N); EI-MS (Relative intensity %): m/z 349.10 (M^+^, 20); Elemental analysis: *Anal*.C_21_H_19_N_3_O_3_: C, 69.75; H, 5.31; N, 11.66; Found C, 69.69; H, 5.20; N, 11.53.

#### 4-((2-furan-2-ylmethylene)hydrazinyl)(1H-indol-1-yl)methyl)-N,N-dimethylaniline (1h)

Light brownish yellow solid; mp 270°C; IR (KBr) (cm^–1^) 3440 (NH str), 3085 (CH-str Ar-ring), 2946 (NH_2_), 1620 (C = N), 1096 (N-CH-N). ^1^H NMR (DMSO-d_6_), δ (ppm) J (Hz): 8.29 (s, 1H, CH = N), 7.93–7.83 (d, *J* = 7.0, 2H, indole), 7.72–6.54 (m, 3H, furan), 7.46–6.68 (m, 9H, Ar), 7.13 (s, 1H, NH), 6.76 (s, 1H, CH), 3.80–3.12 (s, 3H, N-CH_3_); ^13^C NMR (DMSO-d_6_) δ (ppm): 149.17, 128.13, 127.87, 127.80, 112.75, 112.72 (6C, Ph) 149.10, 144.43, 118.93, 112.67 (4C, Furyl ring), 136.57, 128.92, 128.51, 121.75, 120.77, 119.78, 109.57, 100.84, (8C, indole ring), 134.61 (1C, C = N), 41.29 (2C, N-CH_3_) 40.58 (1C, CH); EI-MS (Relative intensity %): m/z 331.37 (M^+^, 20); Elemental analysis: *Anal*.C_22_H_22_N_4_O: C, 73.42; H, 6.20; N, 15.63; Found C, 73.22; H, 6.09; N, 15.43.

#### 2-(furan-2-ylmethylene)hydrazinyl)-3,7-dimethylocta-2,6-dien-1-yl)-1H-indole (1i)

Light brown solid; mp 280°C; IR (KBr) (cm^–1^) 3446 (NH str), 3080 (CH-str Ar-ring), 1620 (C = N), 1094 (N-CH-N). ^1^H NMR (DMSO-d_6_), δ (ppm) J (Hz): 8.30 (d, 1H, *J* = 6.2 Hz, furyl ring), 7.58–7.53 (d, 2H, *J* = 7.0, indole ring), 7.40, 7.31, 7.06, 6.58 (m, 4H, indole ring), 7.12 (s, 1H, NH), 6.90, 6.48 (m, 2H, Furyl ring), 6.68 (s, 1H, CH), 5.47, 5.30 (d, 2H, *J* = 7.7 Hz, citral), 2.00 (d, 2H, *J* = 6.3, citral), 2.04 (d, 2H, *J* = 6.0, citral), 1.82–1.71 (s, 9H, citral);^13^C NMR (DMSO-d_6_) δ (ppm): 149.98, 144.38, 118.97, 112.68 (4C, Furyl ring), 136.52, 128.91, 127.83, 121.68, 120.75, 119.81, 109.65, 100.95 (8C, indole ring), 135.52, 132.02, 123.52, 118.76, 39.45, 26.41, 24.61, 18.63, 16.10 (9C, citral) 134.62 (1C, C = N), 40.60 (1C, N-CH-N); EI-MS (Relative intensity %): m/z 361.22 (M^+^, 20); Elemental analysis: *Anal*.C_23_H_27_N_3_O: C, 76.43; H, 7.54; N, 11.63; Found C, 76.66; H, 7.43; N, 11.42.

#### 1-((2-furan-2-ylmethylene)hydrazinyl)methyl)-1H-indol (1j)

Brown solid; mp 285°C; IR (KBr) (cm^–1^) 3442 (NH str), 3082 (CH-str Ar-ring), 1626 (C = N), 1092 (N-CH-N). ^1^H NMR (DMSO-d_6_), δ (ppm) J (Hz): 8.24 (s, 1H, CH = N), 7.78–6.59 (d, *J* = 6.2 Hz, 3H, Furan), 7.62–7.59 (m, 2H, indole), 7.47–6.45 (m, 9H, Ar), 7.16 (s, 1H, NH), 5.54 (d, *J* = 6.2Hz, 2H, CH_2_); ^13^C NMR (DMSO-d_6_) δ (ppm): 149.17, 145.02, 118.91, 112.62 (4C, Furyl ring), 136.49, 128.98, 127.89, 121.76, 120.72, 119.86, 109.67, 100.91 (8C, indole ring), 134.62 (1C, C = N), 40.59 (1C, CH_2_); EI-MS (Relative intensity %): m/z239.27 (M^+^, 20); Elemental analysis: *Anal*. C_14_H_13_N_3_O: C, 70.29; H, 5.49; N, 17.57; Found C, 70.35; H, 5.78; N, 17.88.

### Biological Screening

#### Cytotoxic Activity

The cytotoxicity experiment was performed according to the United States NCI protocol, previously reported method. A detailed experimental procedure was given in Supplementary Material (Premnath et al., 2015).

#### Molecular Docking

Molecular docking was performed to confirm the molecular interaction with Covid-19 spike core protein to ensure the secondary biological mechanism based on the molecular pose on the binding moiety. The molecular structure of the selected ligand was drawn using Chem. Draw. Before it being considered for molecular interaction, it was 2D optimized by the energy minimization process. The 3D molecular protein crystal structure of spike glycoprotein of SARS-CoV-2 PDB ID 6WPT protein was downloaded. The protein structure was prepared using Schrodinger 12.4 software to remove water molecules and optimize the structure to become suitable to execute flexible docking. In protein preparation, hydrogen atoms were added to increase the hydrophilicity, and already existed co-crystal molecules, and missed loops were optimized (Boyd and Paull, 1995). The ligand preparation module optimized the ligand 3D structure of selected molecules to remove unwanted atomic orientation by molecular and quantum mechanics. Molecular docking, with flexible SP followed by XP, was executed. The grid-based technique, evaluation, and minimization of grid approximation procedure were followed by Premnath et al. (2016) and Muthiah et al. (2020). The confirmation of the best interactive molecule with 6WPT protein was concluded based on the G score and number of hydrogen bonds and bonding efficiency and binding energy.

## Results and Discussion

### Chemistry

**FIGURE 3:**
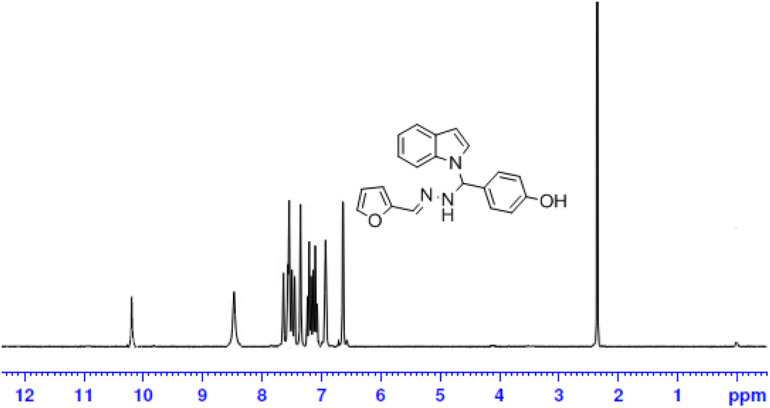
^1^H-NMR spectrum of compound-1c.

**FIGURE 4:**
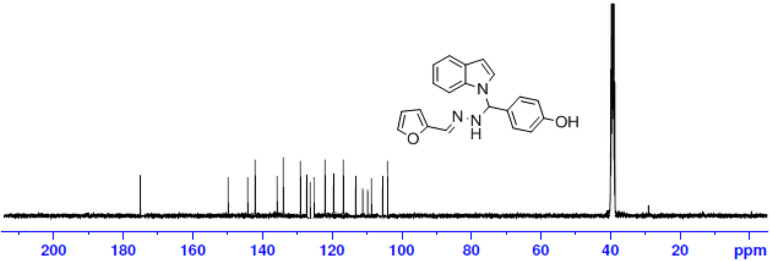
^13^C-NMR spectrum of compound-1c.

**SCHEME 1:**
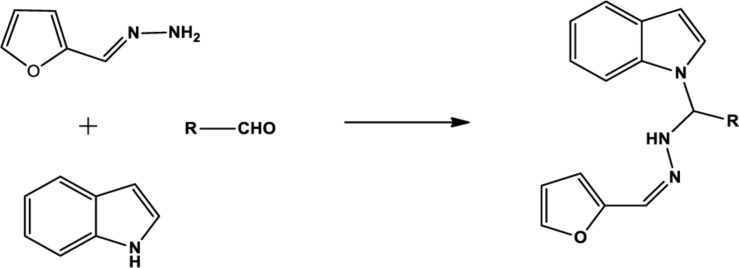
Synthesis of indole derivatives.

The one-pot Mannich reactions of substituted benzaldehyde, indole, and Furan-2-lymethylenehydrzine were done by reflux for 2 h using ethanol, a solvent, without any catalyst. The obtained solid 1-((2furan-2ylmethylene)hydrazinyl)phenyl)methyl)1H-indole (1a) was washed with cooled water and recrystallized using ethanol. It was purified by TLC. Hexane was used as an eluting solvent in TLC. All the synthesized compounds were separated by column chromatography. A similar procedure was carried out to synthesize the other nine compounds (1b–1j) Physicochemical data of synthesized compounds (1a–1j) are given in Table 1. The Figure 3 indicates ^1^H-NMR spectra of compound 1c, and Figure 4 displays ^13^C-NMR spectra of compound 1c. Scheme 1 represents the synthesis of compounds 1a–1j.

**TABLE 1:** Physicochemical data of synthesized compounds (1a-1j).

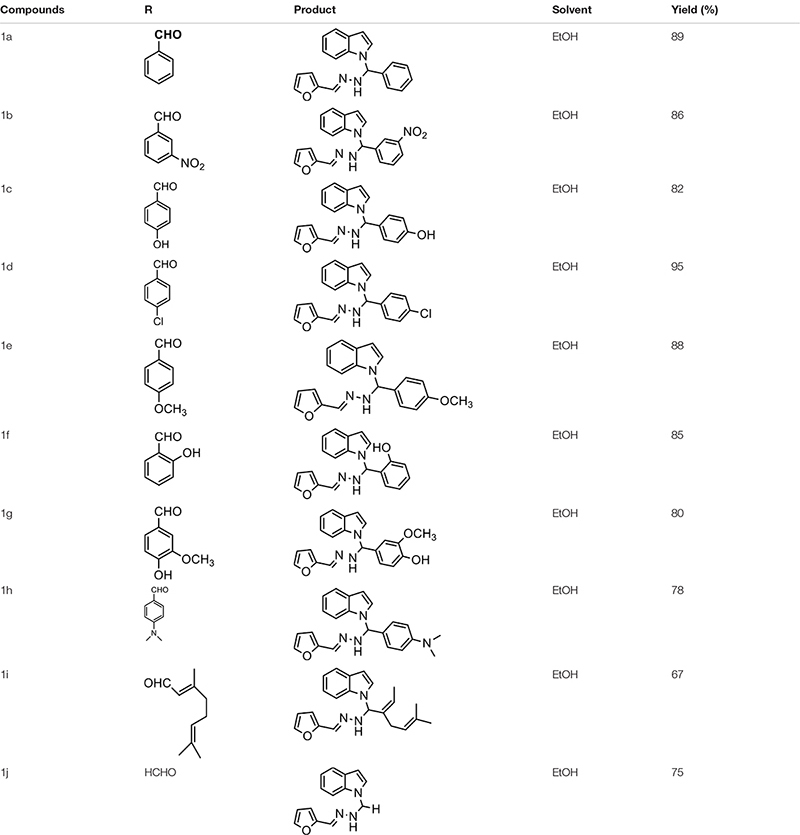

All the newly synthesized indole derivatives were characterized by FT-IR, which showed various functional groups. The ^1^H-NMR spectra of compounds (1a–1j) indicate frequency observed at 7.16–7.07 and 6.79–5.54, corresponding to the NH-CH and CH-Ph protons. The ^13^C -NMR spectra exhibit the peak at 144.42–118.76 and 40.60–40.53, corresponding to the NH-CH and CH-Ph carbon, respectively.

#### Cytotoxic Activity

The newly prepared compounds 1a–1j are examined for their cytotoxic activity according to the United States NCI protocol, which was a previously reported method (Chadha and Silakari, 2017). The 50% growth inhibition (GI_50_), tumor growth inhibition (TGI), and lethal concentration (LC_50_) values were determined. The compounds 1c were a significant activity against (HepG2, LC50-0.9 μm, MCF-7, LC50-0.55 μm, HeLa, LC50-0.50 μm). Doxorubicin was used as a standard drug. None of the tested derivatives had shown significant activity toward the cancer cell lines. The compounds were also evaluated for their possible cytotoxicity in human embryonic kidney cells (HEK-293), lung cells (MRC-5), and liver cells (LO2) by employing MTT assay. The assay results suggested that these compounds did not significantly affect normal kidney cells’ growth (As most of the compound’s IC50 values are >100). Hence, these compounds revealed their safety for the normal cells, and the compound 1c can be taken as lead compounds for further development of more potent agents for HepG2 (Liver), MCF-7 (Breast), HeLa (Cervical) cancer cell lines. The results of cytotoxic screening of compounds (1a–1j) are shown in Table 2, and *in vitro* cytotoxicity of indole derivatives (1a–1j) on normal cells are shown in Table 3.

**TABLE 2:** Cytotoxic activity of synthesized compounds (1a–1j).

Cpds	HepG2			MCF-7			HeLa		
			
	GI_50_ (μm)	TGI (μm)	LC_50_ (μm)	GI_50_ (μm)	TGI (μm)	LC_50_ (μm)	GI_50_ (μm)	TGI (μm)	LC_50_ (μm)
1a	43.2	78.3	>100	–	–	>100	–	–	100
1b	42.2	23.1	56.8	46.1	85.1	>100	51.0	89.2	>100
1c	36.3	65.3	0.9	–	–	0.55	41.3	87.2	0.50
1d	01.0	0.25	54.0	0.89	09.3	65.0	08.9	06.8	0.50
1e	15.9	38.2	44.8	24.4	59.3	66.3	34.2	72.1	>100
1f	29.1	46.8	57.5	18.6	41.8	42.3	21.6	54.7	77.4
1g	48.0	61.3	59.3	34.0	67.4	30.5	37.9	51.9	58.9
1h	21.3	41.0	72.1	12.9	45.3	10.3	52.9	81.3	>100
1i	19.3	28.3	>100	45.6	56.0	59.3	49.0	49.3	55.0
1j	40.3	45.3	66.8	56.0	49.3	78.6	2.3	58.3	48.3
Doxorubicin (standard)	0.01	0.13	0.58	0.02	0.21	0.74	0.05	0.41	0.88

**TABLE 3:** *In vitro* cytotoxicity of indole derivatives (1a–1j) on normal cells^*a*^.

Compounds	MRC5	HEK-293	LO2
	IC_50_ (μm)	IC_50_ (μm)	IC_50_ (μm)
1a	76.36	70.06	67.48
1b	67.21	62.12	72.17
1c	86.66	81.14	87.10
1d	56.25	79.14	66.24
1e	72.76	57.09	56.01
1f	51.24	66.17	68.24
1g	62.61	58.24	70.54
1h	66.32	67.01	58.22
1i	79.41	77.44	66.70
1j	75.14	52.71	70.12

*^*a*^Each compound was tested in triplicate. All error bars represent mean ± SD from three independent experiments.*

None of the tested derivatives had shown significant activity toward the cancer cell lines. The compounds were also evaluated for the possible cytotoxicity in human embryonic kidney cells (HEK-293), lung cells (MRC5), and liver cells (LO2) by employing MTT assay. The assay results suggested that these compounds did not significantly affect the growth of normal cells (as most of the compounds IC_50_ > 100). Hence this compounds revealed their safety for the normal cells and the compound 1c can be taken as lead compound for further development of more potential agent for HepG2 (Liver), MCF-7 (Breast), HeLa (Cervical) cancer cell lines, and *in vitro* cytotoxicity of indole derivatives (1a–1j) on normal cells are shown in Table 3.

#### Molecular Docking

##### PAT binds 6WPT with strong affinity via computer docking studies

**FIGURE 5:**
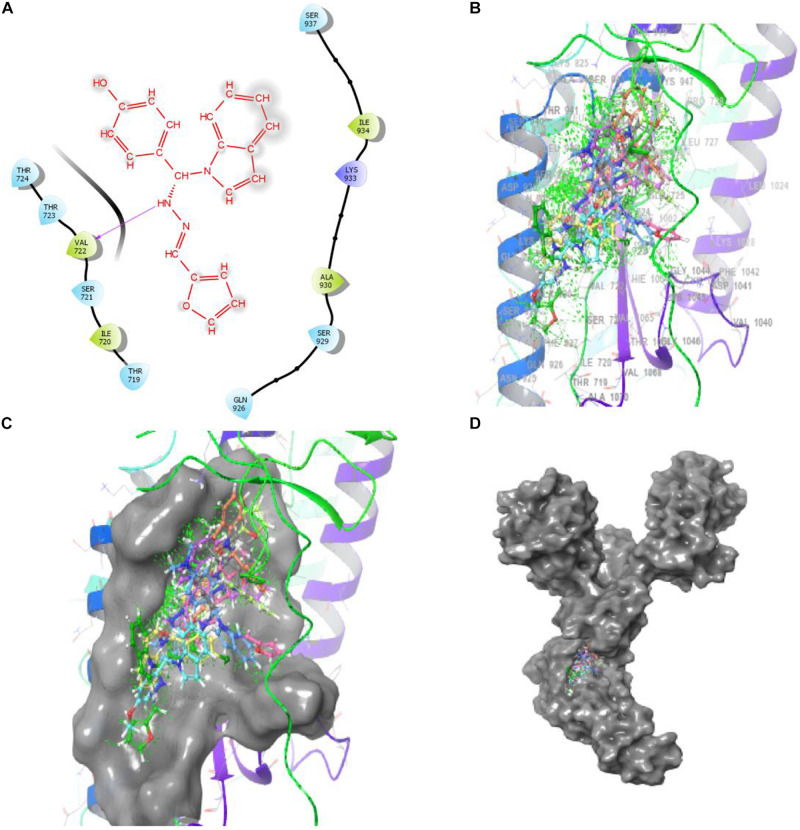
Compound-1c has a high affinity with 6WPT subunit. **(B–D)** Surface protein structure with hydrogen bonding interaction between **(C)** 6WPT interactive molecular pocket and ligand binding with series of ligands using a 3D molecular structure while panel **(A)** shows 2D binding pockets interactive sites.

**SCHEME 2:**
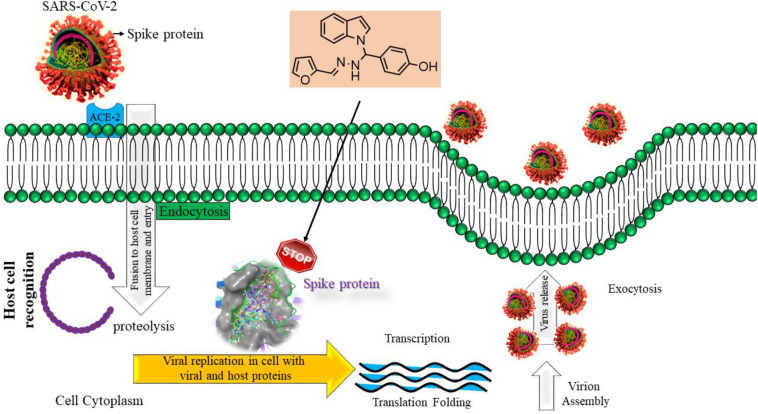
Pathway mechanism of compound-1c interacting with spike protein.

Bimolecular interaction studies were used to characterize the interaction between selected drug-like molecule and protein biomolecular binding sites. The protein interaction study was executed to forecast the interactive visualization modes and binding of small molecule and their respective protein receptors. An investigation of the interactive molecular complex of ligand series disclosed very informative and important connections between the drug like molecular series and the (6WPT) protein receptor. The two-dimensional and three-dimensional protein molecular structural images were perfectly visualized using Schrodinger integrative python software to analyze the molecular interaction between the selective ligand series and protein macromolecule (6WPT) (Figure 5). The overall optimized G score for binding ligands is predicted as −2.808 kcal/mol. This is taken to indicate an expected favorable reaction. Ligand 3 (1c) were perfectly interacted and formed close molecular interactions with amino acid residues on the predicted selective binding sites of VAL367, LEU368, PHE342, GLY339, GLY112, ARG55, LEU47, ASN343, ASP115, TYR32 of receptor protein (6WPT) (XXX) during different biochemical communications of hydrogen bonding, and hydrophobic interaction (Pinto et al., 2020). The binding score of (1c) to 6WPT was moderately burly with a predictable affinity of −2.808 kcal/mol. The docking analysis characterization of novel synthesized molecules are shown in Table 4. Further, ligand series bonded with 6WPT through interactions with hydrogen bonding interaction and Pi-Pi interaction, Pi-Pi stacking interactive protein amino acids side chains of valine (Val), threonine (Thr), serine (Ser), alanine (Ala), and lysine (Lys) were analyzed and predicted important interactive bioactive binding site molecules. The molecular interaction analysis with selected small molecules of (1c) with 6WPT protein was much stronger than other series of selected ligands with a predictable affinity of −2.808 kcal/mol (Figure 5). The pathway mechanisms of spike protein interactions with highly active compound 1c are shown in Scheme 2. All the 2D structures of synthesized compounds are given in Supplementary Materials.

**TABLE 4:** Docking results of synthesized compounds.

Entry Name	Glide g score	Glide e model	Glide energy	XP H-Bond	Bonded Amino acid	Bond length A
3 (1c)	–2.808	–37.395	–29.608	–1.33	VAL 722	2.18
7 (1g)	–2.715	–38.457	–27.458	–0.7	THR 724 ALA 944	1.91 2.08
5 (1e)	–2.174	–28.608	–24.17	–0.641	VAL 722	2.37
2 (1b)	–1.912	–35.298	–30.366	–0.7	LYS 947 ALA 944	2.51 1.80
1 (1a)	–1.636	–31.767	–26.491	–0.7	ALA 944	2.03
6 (1f)	–1.537	–32.323	–25.423	–0.7	THR 724	1.98
4 (1d)	–1.213	–35.839	–28.794	–0.37	SER 937	2.10
8 (1h)	–1.18	–30.149	–29.338	–1.29	THR 724	1.94
9 (1i)	–0.499	–31.625	–26.095	–1.56	THR 724	1.99

#### Structure Activity Relationship

A structure-activity relationship analysis (SAR) was performed to find the link between the chemical structure of a dynamic molecule and its cytotoxic activity. SAR analysis makes it possible to identify the chemical group/atom that plays a critical function in modulating the cytotoxic activity of compounds within the specific system. Using the cytotoxic activity results of the indole Mannich base derivatives, preliminary SARs could be evaluated. The data of the selected indole Mannich base derivatives (1a–1j) showed that compound 1c is the most effective (HepG2, LC_50_-0.9 μm, MCF-7, LC_50_-0.55 μm, HeLa, LC_50_-0.50 μm) control doxorubicin.

Due to the presence of an indole ring fused to a hydroxyl benzaldehyde, it was found that the compound acquires a high cytotoxic activity against cancer cell lines. This was due to the presence of electron releasing hydroxyl group on phenyl ring attached with an indole skeleton. The rest of the compounds demonstrate feeble cytotoxic activity against all the tested cancer cell lines.

Moreover, from the docking results, it can be assumed that the docking score for indole derivatives (1a–1i) have an acceptable range except 1j compound along with essential interaction which can stabilize the compound in the active site of a protein. Compound 1j has no active site because of an absence of electron withdrawing/electron releasing group on it. From the results, the compound 1c has exhibited the highest docking score of −2.808 (Kcal/mol) compared to other compounds.

## Conclusion

We have reported a facile, high-yielding, one-pot procedure for the synthesis of (1a–1j) via Mannich reaction using various kinds of protected aldehydes which was successfully employed and gave very high yields. Moreover, there were no requirements for dry solvents or protective gas atmospheres. All the newly synthesized compounds (1a–1j) were screened for *in vivo* cytotoxicity activities against Hep-G2 (Liver), HeLa (Cervical), and MCF-7 (Breast) cancer cell lines and normal cell lines in Human embryonic kidney cell (HEK293), liver cell (LO2), and lung cell (MRC5). Among the indole derivatives, compound 1c (HepG2, LC_50_-0.9 μm), (MCF-7, LC_50_-0.55 μm), and (HeLa, LC_50_-0.50 μm) was that the most active compound against the Doxorubicin standard. All other compounds were less active against the standard. The synthesized derivatives revealed a high safety level by exhibiting very low cytotoxicity against the normal cell line (HEK-293), (LO2), and (MRC5). Furthermore, we report *in silico* molecular docking studies against SARA-CoV-2 spike proteins and the biological characterization of the results reveal that compound 1c (−2.808 Kcal/mol) has the best multiple biological activities and can be used as a model for future derivatives based on the 1c molecular structure. It may identify the route to develop the best drug against Covid-19.

## Data Availability Statement

The raw data supporting the conclusions of this article will be made available by the authors, without undue reservation.

## Author Contributions

PG: Organic compounds preparation; PP: Preparation of synthetic compound and chemical data analysis; KV: Manuscript editing; MA: Validation; MS: Molecular docking analysis; AI: All kinds of spectral analysis; RS: Investigation and writing original draft preparation through the contributions of all authors. All the authors contributed to the article and approved the submitted version.

## Conflict of Interest

The authors declare that the research was conducted in the absence of any commercial or financial relationships that could be construed as a potential conflict of interest.
