# Corrigendum: Synthesis and Cytotoxic Activity of Novel Indole Derivatives and Their *in silico* Screening on Spike Glycoprotein of SARS-CoV-2

**DOI:** 10.3389/fmolb.2021.716238

**Published:** 2021-08-05

**Authors:** Perumal Gobinath, Ponnusamy Packialakshmi, Kaliappillai Vijayakumar, Magda H. Abdellattif, Mohd Shahbaaz, Akbar Idhayadhulla, Radhakrishnan Surendrakumar

**Affiliations:** ^1^ PG & Research, Department of Chemistry, Nehru Memorial College (Affiliated Bharathidasan University), Puthanampatti, India; ^2^ Department of Chemistry, M. Kumarasamy College of Enginnering, Karur, India; ^3^ Department of Chemistry, College of Science, Deanship of Scientific Research, Taif University, Taif, Saudi Arabia; ^4^ South African Medical Research Council Bioinformatics Unit, South African National Bioinformatics Institute, University of the Western Cape, Cape Town, South Africa; ^5^ Laboratory of Computational Modeling of Drugs, South Ural State University, Chelyabinsk, Russia

**Keywords:** indole, Mannich base, cytotoxic activity, COVID-19, spike protein

The authors Kaliappillai Vijayakumar, Magda H. Abdellattif, Mohd Shahbaaz were not included in the published article and the authors Daoud Ali, Saud Alarifi, and Amal Alotaibi were mistakenly included in the author list. The author list has been corrected throughout the article and in the Author Contributions statement. In addition, the funding information was incorrect and has been amended to include funding for Magda H. Abdellattif. The corrected **Author Contributions**, **Funding** and **Acknowledgments** statements appears below.

The authors apologize for this error and state that this does not change the scientific conclusions of the article in any way. The original article has been updated.

