# Supplementary material for: Synthesis and Cytotoxic Activity of Novel Indole Derivatives and Their in silico Screening on Spike Glycoprotein of SARS-CoV-2
Source: Front Mol Biosci. 2021 May 11;8:637989. doi: 10.3389/fmolb.2021.637989 (PMC8144490; doi:10.3389/fmolb.2021.637989)
Supplement: Supplementary file 1 [file Data_Sheet_1.doc]

Supplementary Material

**Synthesis and cytotoxic activity of novel Indole derivatives and their in *silico* screening on spike glycoprotein of SARS-CoV-2**

Content

^1^H-NMR spectra 2-11

^13^C-NMR Spectra 12-21

Figures-6 22-23

Compound -1a

H^1^- NMR


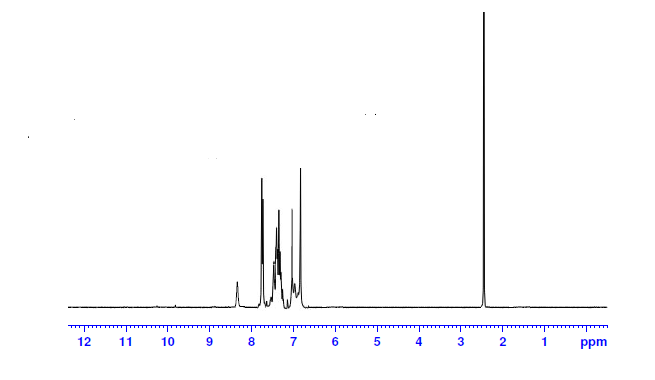


Compound- 1b

H^1^ NMR


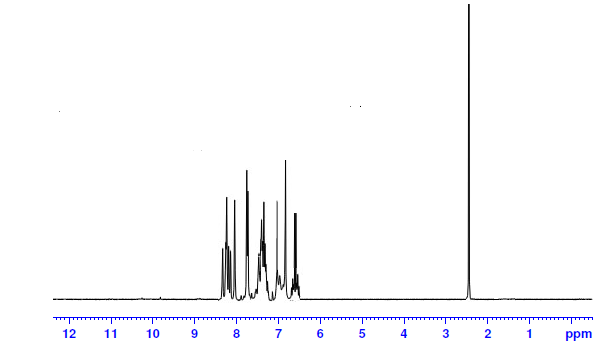


Compound-1c

H^1^ NMR


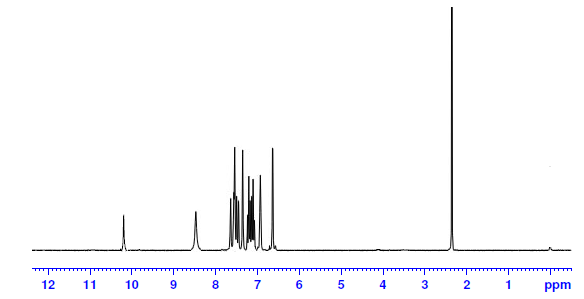


Compound -1d

H^1^ NMR


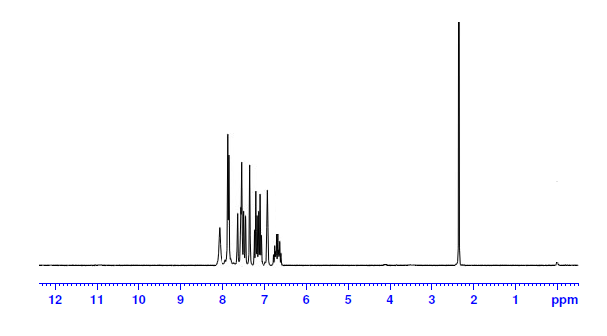


Compound -1e

H^1^ NMR


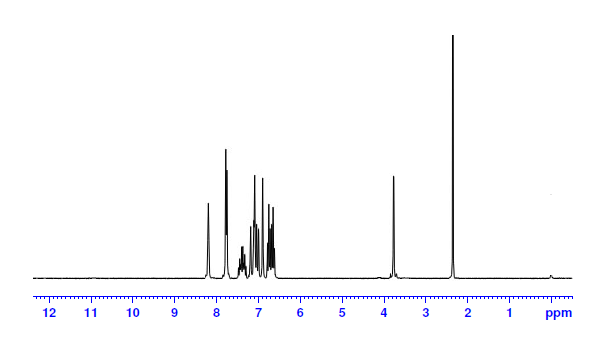


Compound -1f

H^1^ NMR


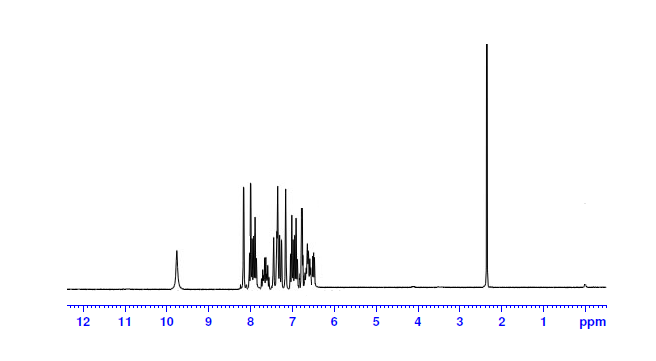


Compound 1g

H^1^ NMR


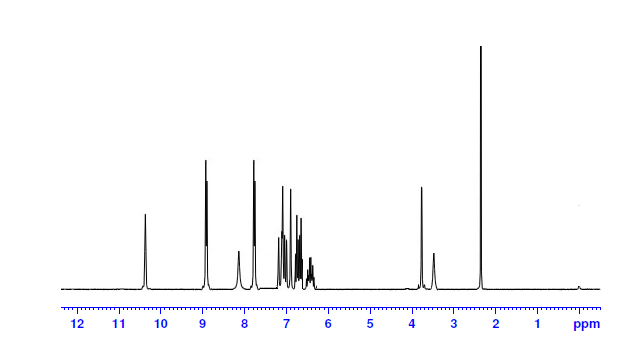


Compound – 1h

H^1^ NMR


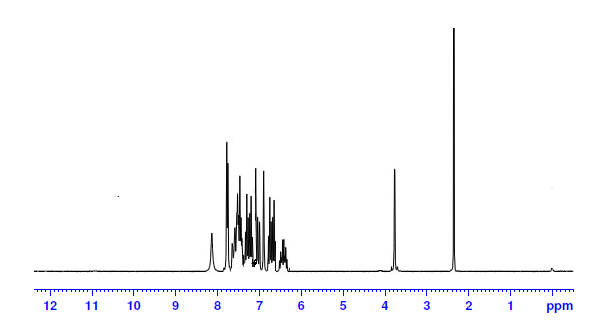


Compound – 1i

H^1^ NMR


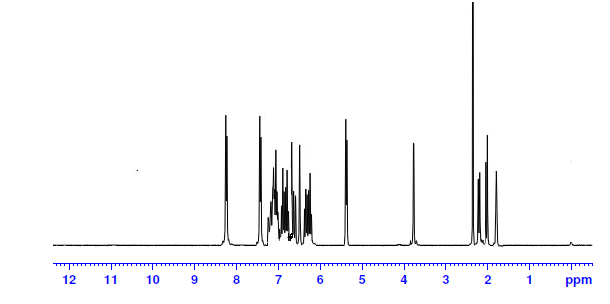


Compound- 1j

H^1^ NMR


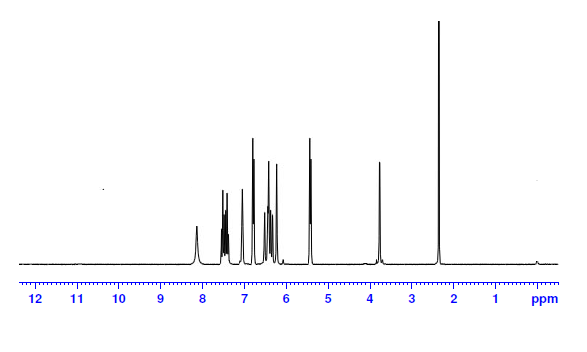


Compound -1a

C^13^ NMR


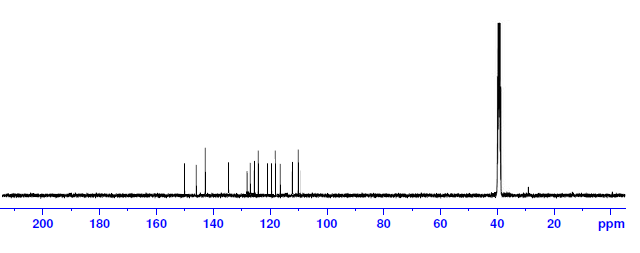


Compound-1b-

C^13^ NMR


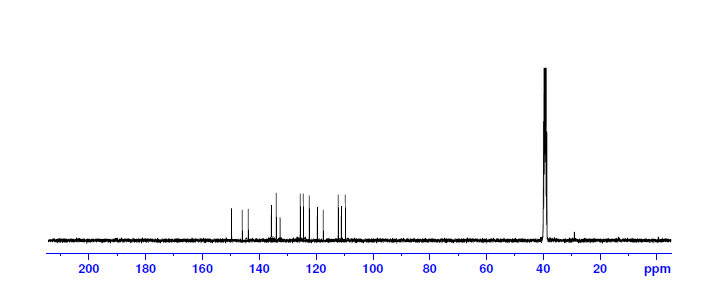


Compound -1c

C^13^ NMR


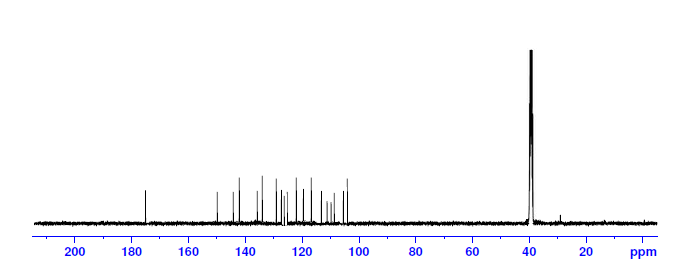


Compound -1d

C^13^ NMR


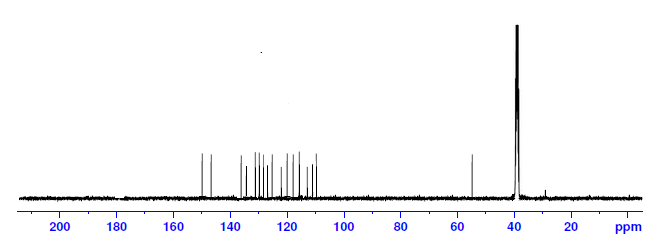


Compound -1e

C^13^ NMR


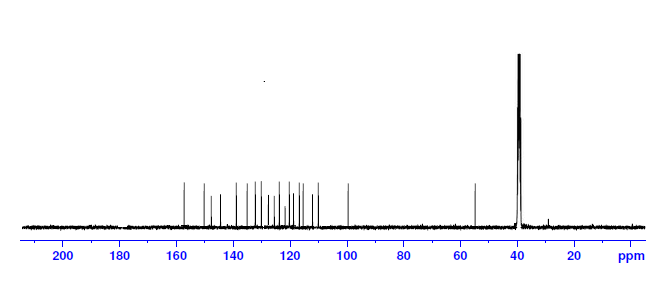


Compound -1f

C^13^ NMR


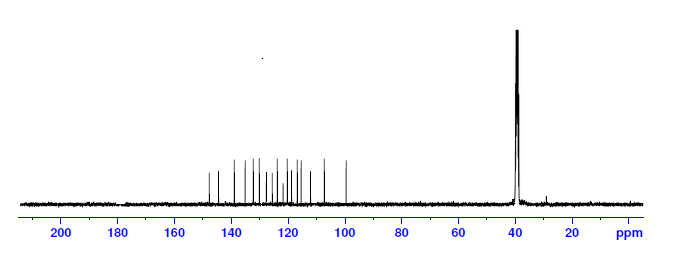


Compound 1g

C^13^ NMR


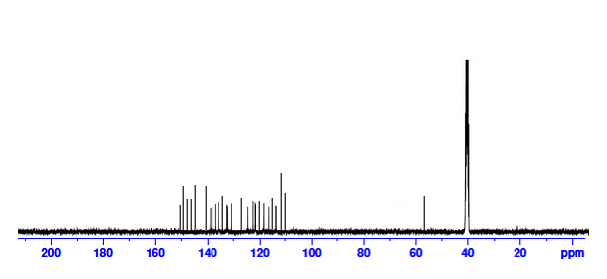


Compound – 1h

C ^13^ NMR


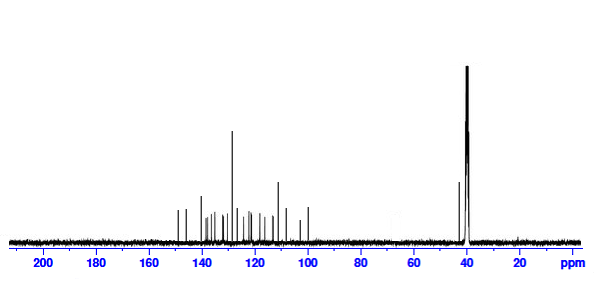


Compound- 1i

C^13^ NMR


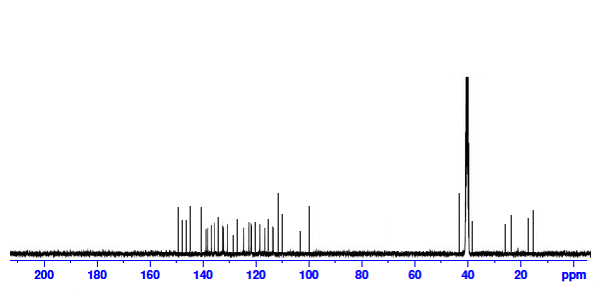


Compound- 1j

C^13^ NMR


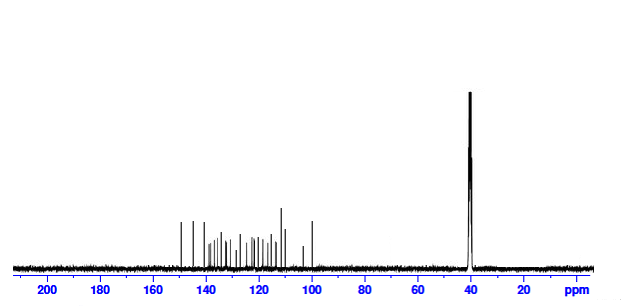


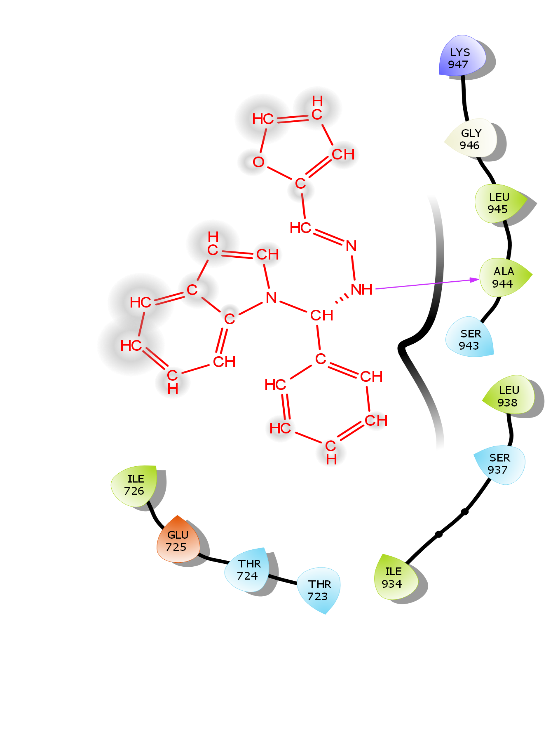

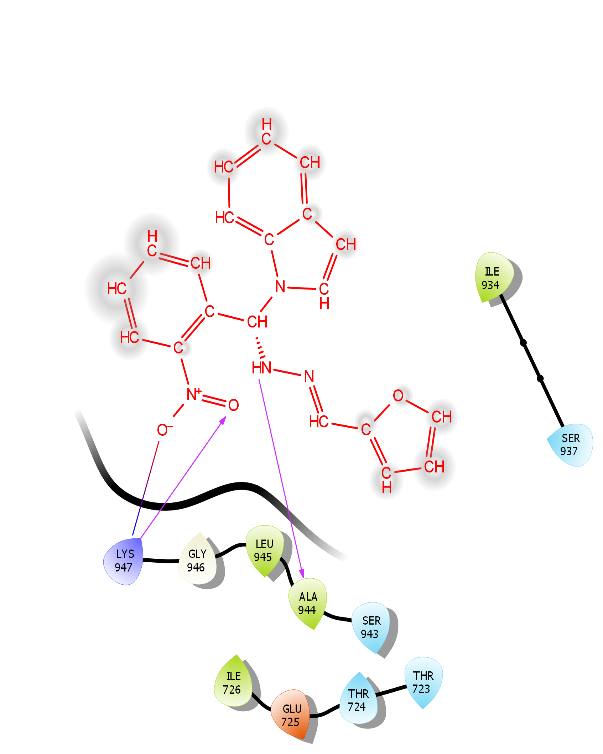


Compound-1a Compound-1b
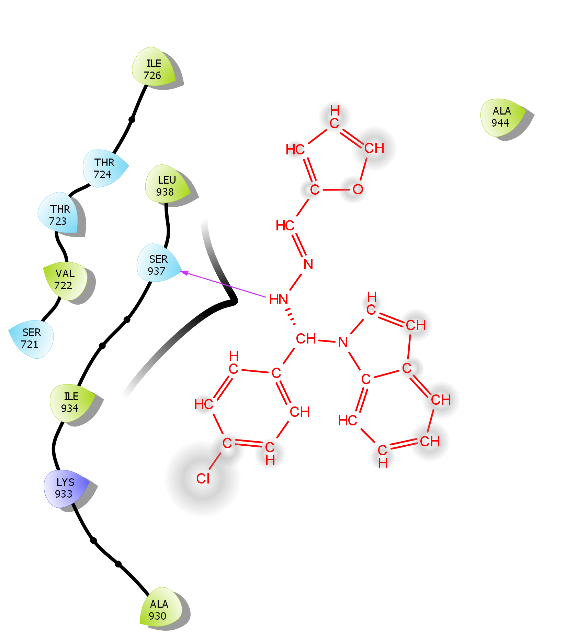

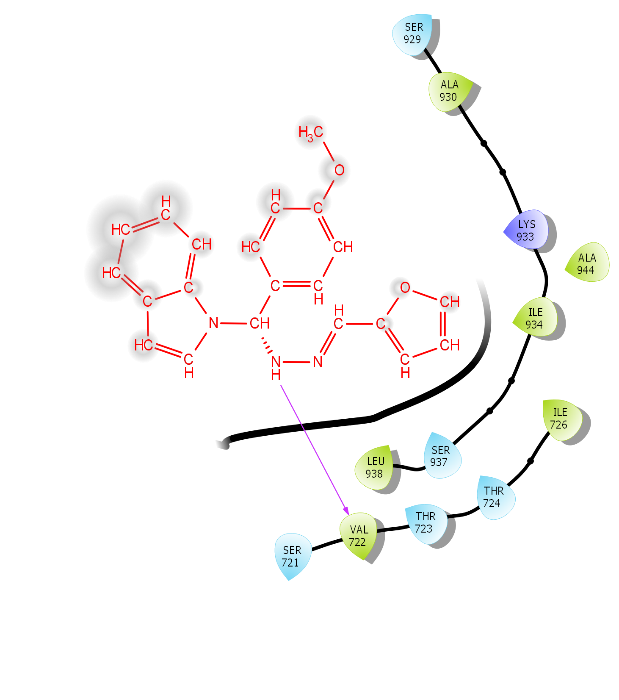


Compound-1c Compound-1e


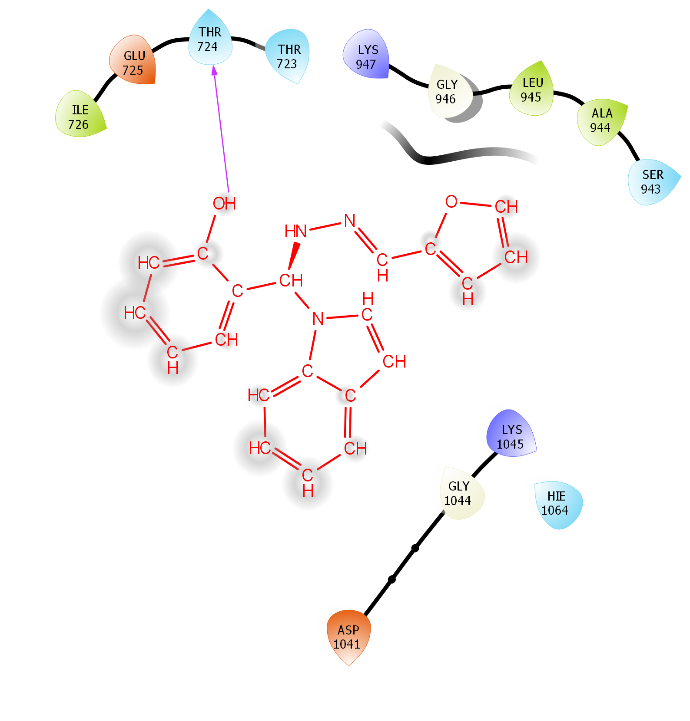

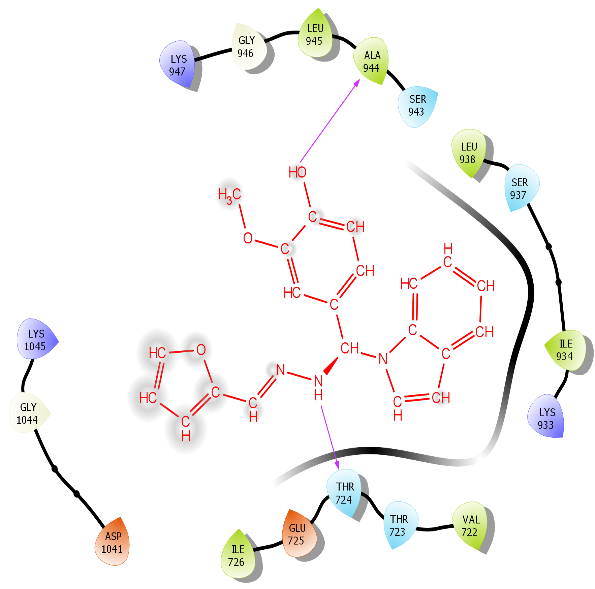


Compound-1f Compound-1g


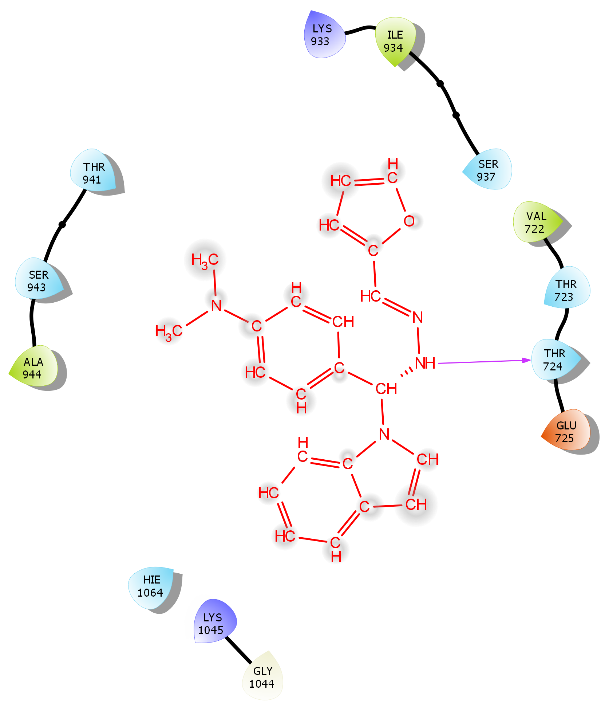

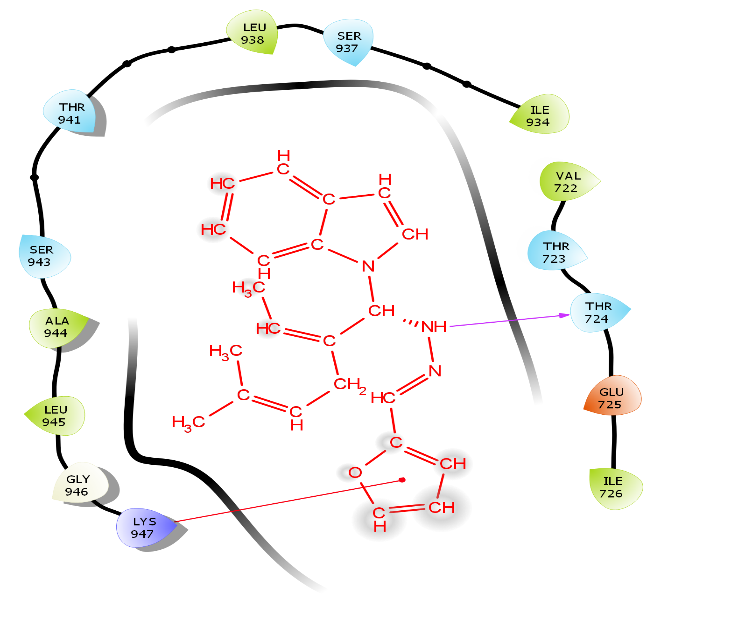


Compound-1h Compound-1i

**Figures 2**: 2d structure of **1a,1b,1c,1e,1f,1g,1h** and **1i**
